# Colorectal cancer and *Blastocystis* sp. infection

**DOI:** 10.1186/s13071-021-04681-x

**Published:** 2021-04-14

**Authors:** Violetta Sulżyc-Bielicka, Lidia Kołodziejczyk, Małgorzata Adamska, Bogumiła Skotarczak, Sylwia Jaczewska, Krzysztof Safranow, Paweł Bielicki, Józef Kładny, Dariusz Bielicki

**Affiliations:** 1 Department of Clinical Oncology, Pomeranian Medical University, Szczecin, Poland; 2 Department of Biology and Medical Parasitology, Pomeranian Medical University, Szczecin, Poland; 3 Institute of Biology, University of Szczecin, Szczecin, Poland; 4 Early Phase Clinical Trial Unit. Maria Sklodowska-Curie Memorial Cancer Centre-Institute of Oncology, Warsaw, Poland; 5 Department of Biochemistry and Medical Chemistry, Pomeranian Medical University, Szczecin, Poland; 6 Department of Radiotherapy, Pomeranian Medical University, Szczecin, Poland; 7 Department of Oncological Surgery, Pomeranian Medical University, Szczecin, Poland; 8 Department of Gastroenterology, Pomeranian Medical University, Szczecin, Poland

**Keywords:** Colorectal cancer, *Blastocystis* sp., ST subtypes, PCR, Light microscopy

## Abstract

**Background:**

*Blastocystis* sp. is a common intestinal protozoan found worldwide. Based on gene analysis, 17 subtypes (STs, ST1–ST17) have been identified, 9 of which have been isolated from humans. Differences in clinical consequences may depend on differences among the STs. Here, we evaluated the prevalence of *Blastocystis* sp. in patients with colorectal cancer (CRC) compared to a control group and assessed the relationships between *Blastocystis* sp. infection and sex; age; and CRC grade, stage, and location.

**Methods:**

The study included 107 CRC patients (41 women and 66 men, median age 65 years); 124 subjects without colorectal cancer or a history of oncological disease comprised the control group (55 women and 69 men, median age 63). Stool samples were collected from patients before oncological treatment and examined using light microscopy (iodine-stained smear). Additionally, PCR-based identification of *Blastocystis* sp. was performed in 95 stool samples from CRC patients and 76 stool samples from the control group.

**Results:**

Light microscopy showed that the prevalence of *Blastocystis* sp. was significantly higher in CRC patients than in the control group (12.15% and 2.42%, respectively; *p* = 0.0041). Multivariate analysis showed that the odds of *Blastocystis* sp. infection were fivefold higher in the CRC group than in the control group. PCR-based molecular examinations demonstrated that the proportion of patients infected with *Blastocystis* sp. was significantly higher in the CRC group than in the control group (12.63% and 2.63%, respectively; *p* = 0.023). The predominant ST in the CRC group was ST3, detected in nine patients (75%), followed by ST1 (2 patients, 16.7%) and ST2 (1 patient, 8.3%). No association was found between *Blastocystis* sp. infection and age, sex, or CRC stage, grade, or location.

**Conclusions:**

The results showed that CRC was associated with an increased risk of opportunistic *Blastocystis* sp. infection, even before oncological treatment. To the best of our knowledge, this is the first report estimating the prevalence of *Blastocystis* sp. infection in CRC patients before oncological treatment in Europe.

**Supplementary Information:**

The online version contains supplementary material available at 10.1186/s13071-021-04681-x.

## Background

The International Agency for Research on Cancer has estimated that 16% of cancer worldwide is caused by infectious factors, including parasites [1]. Colorectal cancer (CRC) is one of the most common neoplasms in humans. Most CRCs are sporadic, and the contributions of environmental risk factors have been widely investigated [2]. Microbes colonizing the gut are also considered potential cancer risk factors [3, 4].

*Blastocystis* sp. is a common parasitic protozoan with a worldwide distribution that is found in the gastrointestinal (GI) tract of humans and a wide range of animal hosts [5, 6]. Its prevalence in humans is estimated to be as high as 10% in developed countries and 50–60% in developing countries [7]. *Blastocystis* sp. is transmitted through the faecal–oral route as well as through contaminated water and food [8, 9]. The pathogenicity of this protozoan is controversial, as it causes non-specific digestive tract symptoms, such as abdominal pain, nausea, vomiting, anorexia, acute or chronic diarrhoea, and weight loss. *Blastocystis* sp. infection is usually associated with alternating episodes of diarrhoea, normal defecation, and even constipation [10]. Poirier et al. suggested an association between *Blastocystis* sp. infection and irritable bowel syndrome (IBS) [11]. On the other hand, a higher rate of *Blastocystis* sp. infection in asymptomatic patients than in those with IBS symptoms was detected in Denmark [12].

The clinical significance of *Blastocystis* sp. infection remains uncertain, mainly because of its common occurrence in both dyspeptic patients and healthy individuals [9, 13]. Some studies have maintained that *Blastocystis* sp. is part of a healthy gut microbiome [13, 14]. However, it has also been reported that *Blastocystis* sp. infection can have features of opportunistic infection, as has been observed in patients with CRC treated with chemotherapy [15].

The identification of this organism at the species level is difficult. *Blastocystis* was originally named *B. hominis*, but subsequent phylogenetic studies limited the name to “*Blastocystis* species” because of the genetic diversity among members within the genus [16]. It was discovered that host specificity and the pathogenic potential of different isolates are correlated with sequence variations in the small subunit ribosomal RNA (SSU-rRNA) gene [17]. Based on these variations, members of the genus have been ordered into several subtypes (STs) [18]. Based on SSU-rRNA gene analysis, 17 STs (ST1–ST17) have been identified, 9 of which have been isolated from humans [19]. Differences in clinical consequences may depend on differences among STs [20]. Because *Blastocystis* sp. are found in both symptomatic and asymptomatic patients, the pathogenicity of this organism remains unclear [21–23]. Some studies have shown an association between *Blastocystis* sp. ST variation and pathogenicity. Dogruman-Al et al. [24] suggested that ST2 is a non-pathogenic genotype of *Blastocystis* sp.

The predominance of *Blastocystis* sp. ST3 among patients with chronic GI illness has been shown in Malaysia [25], Singapore [26], Egypt [27], Turkey [28], the United States [29], and Iran [30]. Khademvatan et al. [20] showed that in southern Iran, the most common ST of *Blastocystis* sp. was ST3, which correlated with the presence of GI symptoms in 44.83% of cases.

Studies evaluating the prevalence of *Blastocystis* sp. in the French population (inhomogeneous population of 788 patients from 11 hospitals) showed that the frequency of *Blastocystis* sp. infection in patients with symptoms of GI disorders was not significantly higher than that in patients without symptoms, and the most common ST was ST3 [31].

The aim of this study was to evaluate the prevalence of *Blastocystis* sp. in patients with CRC compared to that in a control group without colorectal cancer or a history of oncological disease and to assess the relationship of *Blastocystis* sp. infection with the sex and age of the subjects as well as CRC stage, grade, and location. To the best of our knowledge, this is the first report estimating the prevalence of *Blastocystis* sp. infection in CRC patients before oncological treatment in Europe.

## Methods

### Patients

This study included 107 consecutive patients with CRC treated between 2009 and 2014 in the Department of General and Oncological Surgery at Pomeranian Medical University (Szczecin, Poland). There were 41 women (38.3%) and 66 men (61.7%) in the study group, with a median age of 65 years. The control group comprised 124 individuals without CRC or other neoplasms in their medical history, including 55 women (44.4%) and 69 men (55.6%), with a median age of 63 years. Detailed patient characteristics are presented in Table 1. CRC was diagnosed based on colonoscopy examinations conducted in the Department of Gastroenterology at Pomeranian Medical University. Histopathological confirmation of cancer was obtained for all CRC patients. Patients with concomitant neoplasms or with a history of another cancer were excluded from the study. No patient included in the study had previously undergone chemotherapy. Stool samples were taken from CRC patients on the day of admission to the Department of General and Oncological Surgery (before surgery) and were delivered to the Department of Biology and Medical Parasitology at Pomeranian Medical University. The study did not require input from the Bioethics Committee, but the study was approved by the Bioethics Committee of Pomeranian Medical University (No. KB-0012/238/06/18). According to the Bioethics Committee instructions, verbal consent was obtained from patients because of the non-invasive nature of the study. Attestation statements of verbal consent of all participants in the study signed by the physicians were documented in medical records.

**Table 1:** Characteristics of the CRC patients diagnosed with *Blastocystis* sp. infection using light microscopy (LM) (*n* = 107) and PCR (*n* = 95)

Examined parameters	LM *n* = 107	PCR *n* = 95
Age (years)	65	66
Median (range)	(38–88)	(40–88)
Sex, number of patients (%)		
Women	41 (38.3%)	37
Men	66 (61.7%)	58
Tumour location*, number (%)		
Right side	24 (22.4%)	22
Left side	83 (77.6%)	73
Tumour location*, number (%)		
Rectum	45 (42.1%)	40
Colon	62 (57.9%)	55
TNM stage, number (%)		
I	18 (16.8%)	18
IIA	36 (33.7%)	33
IIB	0	0
IIC	1 (0.9%)	0
IIIA	1 (0.9%)	1
IIIB	28 (26.2%)	23
IIIC	7 (6.5%)	6
IV	16 (15%)	14
Astler-Coller stage, number (%)		
A	3 (2,8%)	3
B1	15 (14%)	15
B2	36 (33.6%)	33
B3	1 (0.9%)	0
C1	2 (1.9%)	1
C2	32 (29.9%)	28
C3	2 (1.9%)	1
D	16 (15%)	14
Astler-Coller stage, number (%)		
A + B	55 (51.4%)	51
C + D	52 (48.6%)	44
Grade, number (%)**		
G1	7 (6.7%)	6
G2	78 (74.3%)	68
G3	14 (13.3%)	14
Mucinosum	6 (5.7%)	6
Grade, number (%)**		
G1 + G2	85 (81%)	74
G3 + mucinosum	20 (19%)	20

*Right-side location including the caecum, colon ascendens, and colon transversum. Left-side location including the colon descendens, sigmoid, and rectum. **Lack of grading (G feature) in two patients

### Light microscopy (LM)

Parasitological diagnosis was performed by coproscopy. For the detection of *Blastocystis* sp., the stool samples were examined using iodine-stained smears (×40 magnification).

### Polymerase chain reaction (PCR) analysis

Among the patients with CRC (*n* = 107), PCR examinations of the stool samples for the presence of *Blastocystis* DNA were performed in 95 patients (37 women and 58 men) with a median age of 66 years. Among the control group (*n* = 124), PCR examinations were conducted in 76 patients (26 women and 56 men) with a median age of 64 years. The detailed characteristics of patients with CRC who were PCR-tested for the presence of *Blastocystis* sp. (*n* = 95) are presented in Table 1. DNA extraction from 200 mg of each stool sample was performed using the QIAamp DNA Stool Kit (Qiagen, Hilden, Germany). Before DNA extraction, three cycles of liquid nitrogen/water bath (100 °C) incubation, each for 2 min, were performed to destroy the cyst walls of the protozoans present in the samples. Further DNA extraction was conducted according to the manufacturer’s instructions. For molecular identification of *Blastocystis* sp., nested PCR was performed to amplify a 1.1-kb region of the SSU-rRNA gene using two pairs of primers: SR1F and SR1R for the first reaction [26, 32] and the Forward B and Reverse B primer set for the second reaction (Böhm-Gloning et al. [33], modified by Wong et al. [26]). Each amplification reaction was conducted in a total volume of 10 μL with the following components: 1 μL of DNA, 1× reaction buffer, 2.5 mM of MgCl_2_, 3 pM of each primer, 0.75 nM of each nucleotide, and 0.5 U of polymerase (Allegro Taq Polymerase, Novazym, Poland). For the first reaction, amplifications were conducted as follows: 3 min of initial denaturation at 94 °C; followed by 35 cycles of denaturation at 94 °C for 45 s, annealing at 57 °C for 45 s, and extension at 72 °C for 1 min. The PCRs ended with a final extension at 72 °C for 5 min. For the second reaction, the following conditions were set: initial denaturation at 94 °C for 3 min; 35 cycles of denaturation at 94 °C for 30 s, annealing at 59 °C for 30 s, and extension at 72 °C for 45 s; followed by final extension at 72 °C for 4 min. All PCR amplifications were performed in two replicates in Mastercycler Pro thermal cyclers (Eppendorf, Hamburg, Germany). DNA isolates for positive controls were obtained from stool samples examined previously for the presence of *Blastocystis* sp. using LM. The PCR products were visualized in 1.5% agarose gels stained with ethidium bromide. All samples that were positive for the *Blastocystis* sp. SSU rRNA gene were sequenced (Macrogen, Seoul, Korea) with the Forward B and Reverse B primer sets [26, 33]. The obtained sequences were initially aligned with homologous sequences published in GenBank using BLAST (www.ncbi.nlm.nih.gov) and then using MEGA 6.06 software with ClustalW [34]. Subtype confirmation was performed using the sequence query facility in the *Blastocystis* 18S database (https://pubmlst.org/organisms/blastocystis-spp).

### Statistical analysis

Associations between *Blastocystis* sp. infection, and qualitative variables were evaluated with Pearson’s chi-square test or Fisher’s exact test. Associations of *Blastocystis* sp. infection with age and rank variables (e.g., cancer stage) were analysed with the Mann–Whitney *U* test. Multivariate logistic regression analysis was conducted to identify independent risk factors for *Blastocystis* sp. infection. The threshold for statistical significance was *p* < 0.05. Calculations were performed with Statistica 10 (StatSoft Inc., Tulsa, OK, USA) and Microsoft Excel 2003.

## Results

Statistical analysis did not show differences in sex or age between the CRC and control groups (Table 2).

**Table 2:** Prevalence of *Blastocystis* sp. in the CRC and control groups determined by LM and PCR

Parameter	LM			PCR		
	Patients with CRC *n* = 107	Control group *n* = 124	*p*	Patients with CRC *n* = 95	Control group *n* = 76	*p*
Age, years:						
Median (range)	65 (38–88)	63 (38–88)	0.17^a^	66 (40–88)	64 (38–88)	0.71^a^
Sex, number (%)						
Women	41 (38.3%)	55 (44.3%)	0.42^b^	37%	26%	0.63^b^
Men	66 (61.7%)	69 (55.7%)		58%	50%	
*Blastocystis* sp. infection, patients (%)						
Present	13 (12.15%)	3 (2.42%)	0.0041^b^	12 (12.63%)	2 (2.63%)	0.02
Absent	94 (87.85%)	121 (97.58%)		83 (87.37%)	74 (97.37%)	3^b^

^a^Mann–Whitney *U* test

^b^Fisher’s exact test

*Giardia lamblia* was detected in one examined stool sample, and *Entamoeba coli* was detected in one stool sample. Both cases of parasitic infections were detected in patients with CRC without coexisting *Blastocystis* sp. infection.

### Light microscopy

#### Univariate analysis

The presence of *Blastocystis* sp. was detected in 13 patients with CRC (12.15%) and in 3 individuals in the control group (2.42%). *Blastocystis* sp. infection occurred significantly more often in patients with CRC than in individuals in the control group (*p* = 0.00409; Table 2).

#### Multivariate analysis

Multivariate logistic regression analysis including *Blastocystis* sp. infection, CRC diagnosis, age and sex of all participants (CRC and control groups, together *n* = 107 + 124) as independent variables showed that the odds of *Blastocystis* sp. infection were fivefold higher in the CRC patients than in the control group (odds ratio [OR] 5.41, 95% confidence interval [CI] 1.48–19.74, *p* = 0.010; Table 3). There was no association between *Blastocystis* sp. infection and age or sex (Table 3).

**Table 3:** Logistic regression analysis of the association between *Blastocystis* sp. infection as a dependent variable and sex, age, and CRC diagnosis as independent variables in the CRC and control groups

Independent variables	*Blastocystis* sp. infection diagnosis method			
	LM		PCR	
	OR 95% CI	*p*	OR 95% CI	*p*
Age/1 year of life	1.0085 0.9588–1.0607	0.74	0.9891 0.9353–1.0460	0.70
Sex	1.48	0.49	1.13	0.84
Men vs. women	0.48–4.52		0.35–3.63	
CRC	5.41	0.010	5.40	0.031
CRC vs. control group	1.48–19.74		1.16–25.25	

*Blastocystis* sp. infection was diagnosed using LM (*n* = 107 + 124) and PCR analysis (*n* = 95 + 76)

### PCR analysis

#### Univariate analysis

*Blastocystis* sp. infection was detected in 12 patients with CRC (12.63%) and in two individuals from the control group (2.63%). *Blastocystis* sp. detection by PCR occurred significantly more often in patients with CRC than in the control group (*p* = 0.023).

#### Multivariate analysis

Multivariate logistic regression analysis including *Blastocystis* sp. infection, CRC diagnosis, and age and sex of all participants (CRC and control groups) as independent variables showed that the odds of *Blastocystis* sp. infection were fivefold higher in the CRC patients than in the control group (OR: 5.40, 95% CI 1.16–25.25, *p *= 0.031; Table 3). There was no association between *Blastocystis* sp. infection and age or sex (Table 3). The predominant ST among patients with CRC was ST3, which was detected in nine patients (75%). ST1 was detected in two patients, and ST2 in one patient. ST1 and ST3 were detected in one patient each in the control group (Table 4). The sequences obtained in this study were deposited in the GenBank database under accession numbers MG214872–MG214885. Three of the sequences (accession nos. MG214878, MG214882, and MG214884) had 98.1–100% similarity to the published sequence JQ665862, representing ST1. One sequence obtained in this study (accession no. MG214880) was identical to the published sequence JQ665848, representing ST2. The remaining 10 sequences (accession nos. MG214872, MG214873, MG214874, MG214875, MG214876, MG214877, MG214879, MG214881, MG214883, and MG214885) had 99.4–100% similarity to the published sequence KX618192, representing ST3.

**Table 4:** Prevalence of *Blastocystis* sp. infection detected using LM and PCR with differentiation of STs (CRC group, *n* = 95; control group, *n* = 76)

	LM+ (%)*	PCR+ (%)**	ST1 n+ (%)***	ST2 n+ (%)***	ST3 n+ (%)***
CRC group (*n* = 95)	12 (12.6)	12 (12.6)	2 (16.7)	1 (8.3)	9 (75)
Control group (*n* = 76)	1 (1.3)	2 (2.6)	1 (50)	–	1 (50)

*Positive results on LM, **positive results on PCR, ***positive sample

### Comparison of LM and PCR analysis results

Among the patients with CRC for whom both LM and PCR were performed for diagnosis of *Blastocystis* sp. infection (*n* = 95), *Blastocystis* sp. was detected by LM in 12 patients (12.6%), and 9 of those samples (9.4%) were confirmed by PCR examination (Table 5). In those samples, sequencing identified ST3 (Table 4). In the remaining three samples, *Blastocystis* sp. infection detected by LM was not confirmed by PCR. In 3 of 83 stool samples, the presence of *Blastocystis* sp. was not detected by LM but was detected by PCR (2 samples with ST1 and 1 sample with ST2) (Table 4). In all nine samples positive for ST3 from patients with CRC, *Blastocystis* sp. was also detected using LM. There was a strong positive correlation between the LM and PCR results in the CRC group (phi coefficient = +0.71).

**Table 5:** Comparison of the LM and PCR results for the detection of *Blastocystis* sp. in CRC patients

LM	PCR		
	PCR− (%)	PCR+ (%)	Total (%)
LM− (%)	80 (84%)	3 (3%)	83 (87%)
LM+ (%)	3 (3%)	9 (10%)	12 (13%)
Total (%)	83 (87%)	12 (13%)	95 (100%)

In the subgroup of controls in which both LM and PCR analysis were performed (*n* = 76), the presence of *Blastocystis* sp. was detected by LM in one sample (1.3%), but this was not confirmed by PCR. In two stool samples, *Blastocystis* sp. presence was detected by PCR (1 sample with ST1 and 1 sample with ST3), but it was not detected by LM in either case. Therefore, in the control group, there was no correlation between LM and PCR, as no sample was *Blastocystis*-positive by both methods (phi = −0.02). There were also no associations between *Blastocystis* sp. infection and age, gender, grade, Astler-Coller and tumour-node-metastasis (TNM) stages, location of tumour in the rectum compared to the colon, or left- or right-side location of the tumour according to splenic flexion in CRC patients.

## Discussion

Microbiota alterations, referred to as dysbiosis, are often associated with CRC. Both human studies and studies conducted in animals showed that the gut microbiota related to CRC was distinct from that in subjects without CRC [35–37]. In addition, two meta-analyses of faecal metagenome changes specific to CRC were published in 2019 [38, 39]. The human gut microbiota comprises bacteria, viruses, and eukaryotes (e.g., protozoa, helminths, and fungi). In human CRC samples, cytomegalovirus (CMV), John Cunningham (JC) virus, and human papillomavirus (HPV) have been identified, although the data are conflicting [37, 40]. Additionally, changes in the mycobiome associated with human CRC have been reported [41].

The mechanism of the impact of dysbiosis on CRC carcinogenesis encompasses inflammation, immune regulation, metabolism of dietary components, and genotoxin production [42]. The gut microbiota interacting with the host immune system can affect the inflammatory process in the GI tract [43]. The microbiota produce numerous metabolites significant for human physiology [44]; on the other hand, these metabolites can impact the risk of developing CRC. Another carcinogenic mechanism of the microbiota is the production of DNA-damaging toxins [40, 45, 46]. In a driver–passenger model of CRC, the mucosa of the colon is colonized by pathogenic driver bacteria, which produce genotoxins that induce inflammation and, consequently, the adenoma-carcinoma sequence [47]. On the other hand, opportunistic passenger bacteria might proliferate in CRC tumours and stimulate the infiltration of immune cells [40, 47].

Disruptions in the gut microbiota and changes in its relative abundance can alter the balance, leading to many diseases, including inflammatory bowel disease (IBD) and *Clostridium difficile* infection [48, 49]. Impairment of the symbiotic relationship between the microbiota and the host leads to immune dysregulation and can induce chronic inflammation, resulting in IBD [48]. The microbiota composition varies between certain subtypes of IBD (Crohn’s disease, colitis ulcerosa) and the presence of an active phase [50–52]. The role of gut microbiota alterations in IBD has been widely examined [53, 54]. However, in our study, we focused on patients with CRC, and none of our patients suffered from IBD.

The pathogenic potential of *Blastocystis* sp. remains controversial [55–57]. *Blastocystis* sp. interact with bacterial gut microbes [57, 58]. The increased prevalence of *Blastocystis* sp. is related to changes in the composition of the microbiota in the human host [59–62]. Modifications of the microbiota affect the host immune response [55, 63].

To the best of our knowledge, this is the first report to estimate the prevalence of *Blastocystis* sp. infection in CRC patients before oncological treatment in Europe. Few studies have examined *Blastocystis* sp. infection in CRC patients (those that have were from Uzbekistan, Saudi Arabia, Turkey, and Malaysia) [64–67]. Most studies indicate opportunistic characteristics of *Blastocystis* sp. infection, but there are also reports indicating that *Blastocystis* sp. is a component of the healthy gut microbiome [14].

Our results were obtained from a homogeneous group of patients with CRC before oncological treatment, and individuals in the control group were matched by age to the CRC patients. The results showed that the odds of *Blastocystis* sp. infection patients with CRC were 5 times higher than those in the control group; these results were obtained not only by LM but also by PCR, and the proportion of individuals infected with *Blastocystis* sp. was significantly higher in the CRC group (12.63%) than in the control group (2.63%). Chandramathi et al. [15] demonstrated the opportunistic characteristics of *Blastocystis* sp. infection among patients with CRC (*n* = 15). Our results suggest that CRC is related to an increased risk of opportunistic infection with *Blastocystis* sp. even before oncological treatment, which may have additional effects on the immunological system.

At the time of planning of our study, we could not predict what the results would be, because *Blastocystis* sp. can be considered a marker of a healthy gut microbiota [57]; however, it is not possible to exclude opportunistic *Blastocystis* sp. infection in patients with CRC.

A significantly higher prevalence of *Blastocystis* sp. (80%) was found using LM in 200 CRC patients compared to the control population in Tashkent, Uzbekistan [64]. The prevalence of *Blastocystis* sp. in CRC patients was fourfold higher than that in the control population. However, the authors did not conduct PCR analysis, which could have enabled the determination of the ST of *Blastocystis* sp. [64]. Yersal et al. [66] detected *Blastocystis* sp. in 6.5% of 232 stool samples from cancer patients suffering from different types of cancer (lung, breast, CRC) by LM, but in the CRC patients, *Blastocystis* sp. was detected using PCR in 7.5% of the 66 examined cases [66], among which ST1 was the predominant ST (3 cases, 60%), followed by ST3 (2 cases, 40%). In our study, ST3 was the predominant ST in CRC patients (9 patients, 75%), followed by ST1 (2 patients, 16.7%) and ST2 (1 patient, 8.3%).

Kumarasamy et al. found *Blastocystis* sp. infection prevalence rates of 22.08% in 204 Malaysian CRC patients and 9.95% in the control group [67]. The most common ST was ST3 (12.75%), followed by ST1 (4.41%), ST2 (0.49%), and ST5 (0.49%) [67].

*Blastocystis* sp. infection was confirmed in 74 CRC patients (29.7%) in Saudi Arabia [65], where ST1 was the most predominantly detected ST (54,5%) with a significant risk association (crude OR: 7.548; 95% CI 1.629–34.987; *p* = 0.004).

On the other hand, Beghini et al. [14] showed a lower frequency of *Blastocystis* sp. infection among patients with CRC (5.7%) compared to the healthy control group in their analysis of 12 studies of patient populations from different continents (*n* = 1689) with different diseases of the GI tract, including CRC. It is worth noting that only 53 of those patients suffered from CRC [3, 14].

Differences in the results of studies on the prevalence of various *Blastocystis* sp. STs may be related to the genetic diversity of *Blastocystis* sp., non-homogeneity of the analysed patient groups, and ethnic diversity among the examined populations inhabiting different parts of the world [56].

A possible role for *Blastocystis* sp. in CRC pathogenesis has been suggested [15, 64, 68, 69]. Postulated potential carcinogenic effects of *Blastocystis* sp. infection in humans, especially CRC patients, was examined by Chan et al. [70], and the ability of *Blastocystis* sp. to induce the growth of CRC cell lines by inhibiting the apoptotic effects of CRC cells has been documented [70]. Furthermore, isolated antigens of *Blastocystis* sp. isolates were shown to promote the proliferation of cancer cells via downregulation of host immune cellular responses [22, 68, 70]. Chan et al. [70] showed that antigens isolated from symptomatic human hosts caused a more extensive inflammatory reaction and a higher proliferation rate of CRC cells than isolates from asymptomatic human hosts. Chandramathi et al. [68] also showed that solubilized antigen of *Blastocystis* sp. facilitates growth in the human HCT116 CRC cell line. The antigen isolated from ST3 had the most prominent effect on the proliferation of CRC cells [71], which confirmed the case of severe ST3 *Blastocystis* infection in a 35-year-old man at the time of CRC diagnosis, as described by Padukone et al. [72].

The possible impact of *Blastocystis* sp. infections on CRC carcinogenesis remains unclear. The pathogenicity of *Blastocystis* sp. is suspected to be caused by the release of cysteine proteases by this protozoan. These proteases stimulate mucosal cells to release interleukin 8, which has been associated with gut inflammation [56].

Chronic inflammation is an established risk factor for CRC [73]. Among the molecular mechanisms of CRC pathogenesis, oxidative stress plays an important role and has been shown to be associated with *Blastocystis* sp. infection [69].

In our study, *Blastocystis* sp. infection was confirmed by both LM and PCR in all nine samples positive for ST3 from patients with CRC, but no such confirmation was found for the ST1 or ST2 genotypes. In three CRC patients, *Blastocystis* sp. infection detected by LM was not confirmed by PCR, possibly due to the presence of other organisms in the stool samples and non-specific amplification, which was revealed by sequencing of the obtained PCR products. A possible explanation for this discrepancy could also be a misdiagnosis of the parasite by light microscopy.

However, there was a strong positive correlation between the LM and PCR results in the CRC group (phi coefficient = +0.71). The presented results show that LM is an inexpensive, sensitive, and accessible method in daily practice. On the other hand, the PCR method allows for the identification of STs that may have different pathogenic potentials.

## Conclusions

We demonstrated that the prevalence of *Blastocystis* sp. was higher in CRC patients than in the control group, independent of the diagnostic method used. In addition, ST3 was the predominant *Blastocystis* sp. ST among Polish CRC patients. Furthermore, *Blastocystis* sp. infection occurred five times more often in the CRC group than in the control group, independent of age, sex, and diagnostic method. No association was found between *Blastocystis* sp. infection and age, sex, staging, grading, or CRC location. Together, these results show that CRC is associated with an increased risk of opportunistic *Blastocystis* sp. infection even before oncological treatment. The potential relationship of *Blastocystis* sp. with CRC carcinogenesis needs further study (Additional file 1).

## Supplementary Information

**Additional file 1.** Database.

## Data Availability

All data generated or analysed during this study are included in this published article and attached in the “Sulzyc-Bielicka-database xls” file.
